# The Daily Mile: 15 Minutes Running Improves the Physical Fitness of Italian Primary School Children

**DOI:** 10.3390/ijerph16203921

**Published:** 2019-10-15

**Authors:** Paolo Riccardo Brustio, Anna Mulasso, Danilo Marasso, Camilla Ruffa, Andrea Ballatore, Paolo Moisè, Corrado Lupo, Alberto Rainoldi, Gennaro Boccia

**Affiliations:** 1 NeuroMuscular Function Research Group, School of Exercise and Sport Sciences, Department of Medical Sciences, University of Torino, 10143 Turin, Italy; anna.mulasso@unito.it (A.M.); corrado.lupo@unito.it (C.L.); alberto.rainoldi@unito.it (A.R.); gennaro.boccia@unito.it (G.B.); 2 School of Exercise and Sport Sciences, SUISM, University of Torino, 10129 Turin, Italy; danilo.marasso@unito.it (D.M.); cami.ruffa@gmail.com (C.R.); andrea.ballatore@rocketmail.com (A.B.); paolo.moise@unito.it (P.M.)

**Keywords:** active break, school-based intervention, health promotion, healthy lifestyle, physical activity

## Abstract

The Daily Mile™ is an innovative school-based intervention that requires children to run or jog outside for 15 min at a self-selected pace during class time. Today, only one study has investigated the efficacy of The Daily Mile on physical fitness, which was conducted with Scottish children. Thus, we aimed to evaluate the feasibility and effectiveness of The Daily Mile in Italian primary schools. A total of 486 children participated in The Daily Mile for 3 months (experimental group), whereas 309 children continued their daily school routine (control group). The 6-min run test, standing long jump, body mass index, and waist-to-height ratio were assessed. Their teachers completed surveys for assessing the intervention acceptability. After correction for age and gender, significant group × time interactions were observed in the 6-min run test and standing long jump results. In the post-test period, the experimental group showed improvement in the 6-min run test and standing long jump results. Overall, the teachers were satisfied with the program and found it suitable for their school context and easy to implement. The Daily Mile was successfully implemented and smoothly accepted in the day routine of Italian primary schools.

## 1. Introduction

International physical activity guidelines support and recommend that children spend a minimum of 60 min each day in moderate-to-vigorous-intensity physical activity (MVPA) [1,2,3]. However, approximately 60% of children fail to meet these guidelines [1,4,5]. For example, a recent study in eight European countries showed that only 9% of girls and 26% of boys were physically active and performed ≥60 min/day of MVPA [6]. Moreover, the prevalence of obesity and overweight among European children (aged 6–9 years) ranged from 18.4% to 49% in both sexes [7]. In Italy, these trends are even more alarming; in 2016, approximately 30.6% of Italian children aged 6 to 10 years were classified as obese or overweight and presented a lower level of MVPA compared to other European children (2.6% and 9.5% of girls and boys, respectively) [8].

Promoting opportunities to increase physical activity and MVPA, in particular, is an important issue for health promotion, especially throughout childhood [9]. It may help prevent and/or reduce the incidence of overweight or obesity [9] and chronic diseases in adulthood [10]. The school context is a favourable place to promote many forms of physical activity [11,12,13] and to prevent overweight [14] and fitness decline [15,16,17] in childhood and adolescence. Furthermore, school-based policy initiatives can promote physical and health literacy for a lifelong healthy and active lifestyle, essential components to create a more active society [18].

Recently, approximately half of Scotland’s primary schools introduced a physical intervention called The Daily Mile™ to increase the opportunity to perform MVPA in the primary school context. The Daily Mile is a physical activity program, which requires children to run or jog outside for 15 min (approximately 1 mile) each day at a self-selected pace during class time. The first primary school study (children aged 4–12 years) on The Daily Mile found a relative increase in MVPA (approximately 9 min per day), together with a decrease in sedentary behaviour (approximately 18 min per day) [15]. The authors also found an improvement in fitness levels, evaluated using the 20-m shuttle run test, as well as in body composition, measured by skinfolds, after 8 months of intervention [15].

The Daily Mile is growing in popularity throughout the United Kingdom and Europe and in some schools in the USA (https://thedailymile.co.uk/). However, it is essential to test the efficacy of The Daily Mile in different educational contexts [15]. For this reason, we aimed to understand whether this program can be feasible and effective in Italy. Notwithstanding, no studies have investigated the effect of The Daily Mile in Italian primary schools.

Therefore, the aim of this study was two-fold: (1) to assess the adherence, feasibility, safety, and acceptability of The Daily Mile by teachers; (2) to quantify the effectiveness of The Daily Mile in terms of physical fitness and body composition in a sample of Italian primary school students.

## 2. Materials and Methods

### 2.1. Participants

This quasi-experimental, baseline/post-test study included an experimental group, which adopted The Daily Mile (described below) in the school day routine, and a control group, which received the usual daily school activity. Specifically, the children in the control group did not perform any scheduled physical activity outside the curricula. They exclusively followed the curricula hours of physical education lessons according to national guidelines (CM 10-9-1991, n. 271). Differently, the experimental group performed The Daily Mile in addition to the curricula hours of physical education. Consequently, the difference between control and experimental group is the idea of allowing the children in the experimental group to jog and/or run independently for 15 min. All participants were assessed 3 months apart. The study was conducted from March to May 2018.

A convenience sample of schools in the neighbourhood of Turin (North Italy) participated in the study. Information sessions were held in all recruited schools to inform the children’s teachers and parents/guardians regarding the aim of the study. After these information sessions, the teachers voluntary decided to introduce (experimental group) or not to introduce (control group) The Daily Mile in their usual daily school activity or to decline the study participation.

Five primary schools (including a total of 45 classes) participated in the study. All schools represented the standard Italian school. They belonged to the same institutional and regional office (i.e., Ambito Territoriale di Torino), had similar facilities, and used identical educational curricula. Therefore, any possible potential variances in education delivery impacting the outcome measurements were reduced [15].

Students with an intellectual or physical disability, and those who did not participate in baseline data collection were excluded from the study. However, some students with disabilities were included in the experimental group and, therefore, participated in The Daily Mile. All teachers involved in the program were included in the study.

The parents/guardians and teachers of the students provided written informed consent for participation in the study, according to the ethical standards provided in the 1964 Declaration of Helsinki. Additionally, a student’s consent form was included. The study was approved by the Ethical Committee of the University of Torino (protocol number: 203427).

**Figure 1:**
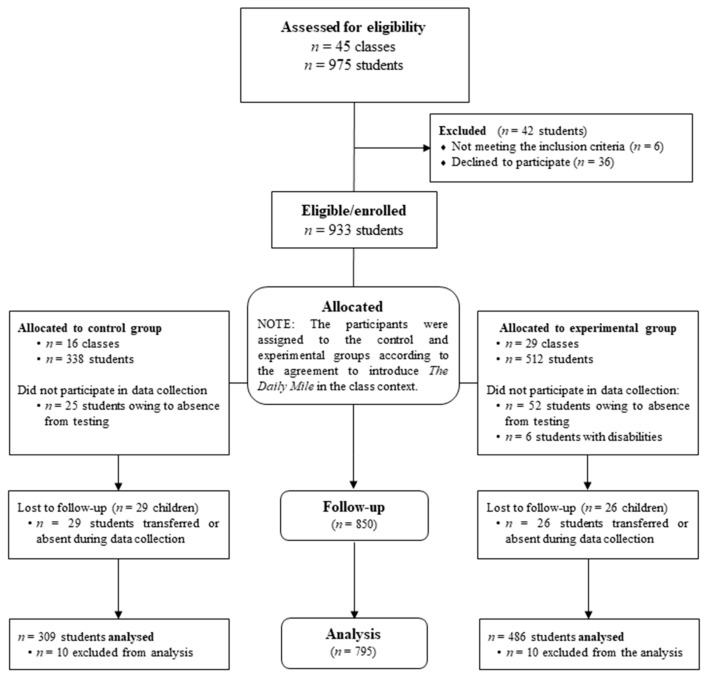
Recruitment process of the study participants.

The classes were divided and labelled as those that agreed to introduce The Daily Mile (experimental group) and those that did not intend to introduce The Daily Mile (control group). The experimental group consisted of 486 students (mean age, 8 ± 1 years; 45.7% were girls), for a total of 29 classes. The control group included 309 students (mean age, 8 ± 1 years; 49.8% were girls), for a total of 16 classes. Thirty-three teachers participating in the study (mean age, 45 ± 5 years; 100% were women) were included. For more details on the recruitment process, see Figure 1.

### 2.2. Procedure and Instrumentation

The Daily Mile intervention consisted of running or jogging at a self-selected pace for approximately 15 min outside the school buildings [15,19,20,21]. Children were instructed to run or jog for the full 15 min and, if necessary, to only occasionally stop for resting [22]. The classroom teachers conducted the activity for 3 months (from March to May 2018). Although the teachers could decide not to conduct the activity because of unfavourable weather, they were encouraged to continue regardless of the weather. No indoor activities were performed. The activity was performed during class time and did not replace normal physical education lessons and breaks. The teachers were free to perform the activity during the school day when it best suited their own timetable. The research team met the teachers of all schools to explain the purpose of the intervention, to provide basic advise, and to deliver a leaflet produced by https://thedailymile.co.uk/ [22] about the school implementing of The Daily Mile. According to Chesham and colleagues [15], no additional instructions were given.

Baseline and post-test assessments of the height, weight, waist circumstance, and 6-min run test and standing long jump results were performed by the same trained and qualified investigators. The children underwent the measurements in a random order in one session lasting between 1 and 2 h according to class size and school timetable.

Height was measured using a portable stadiometer (Model 214; Seca, Hamburg, Germany) with an accuracy of 0.01 m. Weight was measured using an electronic scale (Model 876; Seca, Hamburg, Germany) with an accuracy of 0.1 kg. Both height and weight were measured without shoes. Waist circumstance was measured to the nearest 0.01 m in the midway between the lowest rib and the iliac crest with the participants in the standing position. The Body Mass Index (BMI) was calculated as body mass divided by height squared (kg·m^−2^). The waist-to-height ratio was calculated by dividing waist circumstance by height.

The 6-min run test was used to assess aerobic fitness [23,24]. This test requires participants to run as far as possible in 6 min. The distance covered by each participant was measured in metres [23,24]. The intraclass correlation coefficient of this test was 0.86 in children aged 6 to 10 years [23]. The standing long jump was used for assessing muscle power of the lower limb [23,24]. The subjects were instructed to stand in a high starting position with their feet behind the starting line and arms hanging loose to the side and to jump as far as possible in a horizontal direction [23,24]. This test measures the distance jumped. The distance of the jump is measured to the nearest 0.01 m. The reliability of the test in children aged 6 to 10 years was 0.96 [23].

The teachers monitored the adherence of their class to the program and recorded any adverse event (injuries or accidents) that occurred during the activity. Moreover, at the post-test assessment, the teachers filled a survey developed in accordance with feasibility studies guidelines [25] to investigate the perceptions on the feasibility of The Daily Mile (see Table 1). The survey investigated the following six outcomes mostly related to the acceptability of the program [25]: perceived positive or negative effects on participants or organisation, perceived appropriateness, easy or difficult factors affecting implementation, resources needed for implementation, satisfaction, and intention to use. This original survey was written in Italian and translated into English for publication purposes. For each item, a five-point Likert scale was used [from a minimum of 1 (strongly disagree) to a maximum of 5 (strongly agree)]. The response options were categorised as Favourable, Neutral, and Not Favourable to The Daily Mile.

### 2.3. Data Analysis

Extreme outliers for outcome variables, defined as above or below 3 standard deviations from the mean, were identified and excluded from the analyses. Data distribution was examined using the Shapiro–Wilk test. The assumption of normality for the outcome variables in the experimental and control groups was confirmed (*p* > 0.05). Demographic data and baseline test performance were compared between the groups using *t*-test analysis (quantitative variables) or chi-squared test (qualitative variables) to exclude significant differences at baseline.

The difference between experimental and control groups over the time was investigated using linear mixed models with BMI, waist-to-height ratio, and 6-min run test and standing long jump results as separate dependent variables. Group (i.e., experimental and control groups) and time (i.e., baseline and post-test) were considered as fixed effects, with classes and children identification included as a random effect within the model. Specifically, for the random effect, the children were nested within classes. By using the BMI as a dependent variable, the fitted mixed model was singular. Thus an additional model was used for this dependent variable with group and time as fixed effects and only children’s identification as a random effect. In the above linear mixed analysis, age and gender were included as covariates in the models.

Differences between the experimental and control groups were determined by significant group × time interactions. Analysis of frequencies was performed for the teachers’ survey. The level of significance was set at *p* = 0.05. The Statistical Package R (version 3.5.2, R Foundation for Statistical Computing, Vienna, Austria) with the packages lme4 (version 1.1.19) [26] and emmeans (version 1.3.2) [27] and the Statistical Package for Social Sciences (SPSS Inc., version 24.0 for Windows, SPSS, Chicago, IL, USA) were used for all statistical analyses.

## 3. Results

Figure 1 summarises the recruitment process of the study population. Approximately 91.7% of eligible students were included in the study, and a total of 93.5% completed the baseline and post-test sessions (91.4% and 94.9% of the students in the experimental and control groups, respectively). A total of six students with disabilities participated in The Daily Mile but were not evaluated for outcome variables. All participants completed the demographic data assessments and baseline evaluations of BMI, waist-to-height ratio, and standing long jump results. Only 481 of the included students (60.5%) completed the 6-min run test (70.7% and 47.2% of the students in the experimental and control groups, respectively) because of the unavailability of the track on the day of the measurements.

Briefly, no significant difference was observed between the experimental and control groups in terms of gender, BMI, and standing long jump results (all *p* > 0.05). Conversely, the control group had a slightly older age (t_793_ = 3.043, *p* = 0.002) and showed a lower waist-to-height ratio (t_790_ = 0.626, *p* = 0.007) and higher 6-min run test results (t_479_ = 5.097, *p* < 0.001) than the experimental group.

On average, The Daily Mile was implemented three times per week. No adverse events or accidents occurred during the activities. Table 2 shows the estimated mean values and 95% CI of the experimental and control groups at baseline and the post-test.

Significant group × time interactions were observed in the 6-min run test (F_1,532.22_ = 5.011, *p* = 0.026) and standing long jump results (F_1,790.42_ = 3.877, *p* = 0.049). In the 6-min run test, the experimental group showed an increased result between baseline and the post-test (estimated difference = 25.15 m, standard error (SE) = 6.39 m, *p* < 0.001; percent change = 3.1%) compared with the control group (estimated difference = 4.44 m, SE = 6.69 m, *p* = 0.911; percent change = 0.5%). Moreover, in the standing long jump, the experimental group showed an increased result between baseline and the post-test (estimated difference = 5 cm, SE = 0.6 cm, *p* < 0.001; percent change = 4.7%) compared with the control group (estimate difference = 3 cm, SE = 0.8 cm, *p* = 0.001; percent change = 3.8%). On the contrary, no significant group × time interactions were observed in BMI (F_1,793_ = 0.792, *p* = 0.374) and the waist-to-height ratio (F_1,793_ = 0.395, *p* = 0.529).

Table 1 reports the teachers’ responses to the feasibility survey. Most teachers (81–96%) perceived positive effects on the school environment (Section 1). Approximately half of the teachers (43–59%) perceived the program as appropriate for the school context, whereas 34–53% were neutral with regard to this (Section 2). Except for controlling the class during the activity (neutral responses: 44%; favourable: 47%), most teachers (62–93%) found The Daily Mile easy to implement under many standpoints (Section 3). Most teachers (78–90%) found that The Daily Mile did not require the allocation of organisational or personal resources; however, 18–33% of them would possibly prefer external support (Section 4). In general, 72% of the teachers were satisfied with the activity (Section 5), and half of them would repeat the program next school year (Section 6).

## 4. Discussion

This study investigated the effectiveness and feasibility of The Daily Mile in Italy. The main results of our study were that participation in The Daily Mile during school days had a positive impact on physical functions and that the program was feasible in the Italian context. The activity was successfully implemented three times per week on average. No adverse events or accidents occurred during the activities, suggesting that The Daily Mile was safely implemented. Moreover, although it was not possible to assess disabled children, the study showed that The Daily Mile can be an inclusive activity for everyone.

School-based interventions may contribute to increasing daily physical activity, reducing sedentary behaviours per day, and to improving the physical fitness of school-aged children [9]. Compared with the control group, the experimental group showed improvements in the 6-min run test and standing long jump results, but no differences in BMI and waist-to-height ratio. The present results showed an improvement in the aerobic fitness of the experimental group compared to the control group, as suggested by the increase of about 25 m, corresponding to 3.1% in the 6-min run test (Table 2). This was the main physical outcome of the study, which was an expected result. Indeed, adopting the same intervention in the Scottish context, Chesham and colleagues [15] found an improvement in aerobic fitness evaluated using the 20-m shuttle run fitness test. Specifically, the authors found an improvement of 39 m in shuttle distance after 8 months of intervention (about 5%). Herein, we adopted and conducted the program for 3 months, which may explain why we found a relatively small increase in physical fitness. However, considering the limited amount of time dedicated to the activity, this can be considered a positive finding. This finding is particularly important because, as a simple activity (i.e., running or walking for 15 min), it may counteract the decreasing trend in aerobic fitness observed in children [28].

We also found an improvement of 4.7% in the standing long jump (Table 2), possibly suggesting an increased lower body muscle strength. This was an unexpected result; indeed, running or jogging does not specifically target muscle strength or power qualities. However, previous studies have already suggested that increasing the amount of physical activity improves lower body muscle strength in school-aged children [29,30]. Accordingly, we may speculate that beyond growth and maturation, children participating in The Daily Mile showed an improvement in lower body muscle strength.

On the contrary, no improvement in BMI and waist-to-height ratio was observed (see Table 2). This was in line with the finding of a previous meta-analysis showing no improvement in BMI after school-based physical interventions lasting for a minimum of 6 months [31]. Notwithstanding, recent long-term school-based physical interventions (ranging from 12 to 72 months) have been reported to promote a better BMI [32]. In the Scottish Daily Mile, the authors did not observe any change in BMI, but a reduction in adiposity measured by skinfolds 8 months after the introduction of the activity [15]. Thus, the lack of reduction in BMI may be likely due to the restricted duration of the intervention.

In terms of acceptability, the teachers reported a general overall positive favour to the activity (Table 1), according to previous studies [20,21]. The Daily Mile was easy to implement; indeed, most teachers (91%) reported that the activity did not require many resources to be performed. Further 78% of them stated that the organisation was easy to manage, and none of them reported any negative effects on the school context (approximately 97%). A large acceptability of the activity has also been reported in terms of appropriateness for inclusion in the school context. The teachers’ perception of The Daily Mile regarding the management of the class (e.g., to control the children during the activity and to move the group outside the school buildings) was favourable. Furthermore, the majority of the teachers perceived the activity as safe (approximately 91%), and none of them found the activity difficult to perform (Table 1). Indeed, only 0% to 7% of the teachers reported some minor criticisms in one or more of the aspects mentioned above. Only a few teachers (<7%) reported minor criticisms regarding the interruption and restarting of the teaching activities, and none of them reported any negative effect on their teaching activity. These findings are in contrast with a similar study in Scotland that reported an increased pressure on teachers’ workload, as well as their concern about learning time [16]. However, our results underlined an important finding, since the amount of time allocated to physical activity in school settings tends to also be restricted because of teachers’ concerns regarding the perceived lack of available time and the perception that physical activity may threaten academic achievement [33,34,35]. Our finding suggests that the teachers’ potential concerns regarding academic activities are almost fully overtaken once the activity becomes a part of the daily routine. Overall, the teachers were satisfied with their participation in The Daily Mile (approximately 72% of positive responses). It was not obvious that this initiative was suitable to the Italian socio-cultural peculiarities. However, only half of the teachers would like to participate in the program in the next academic year (approximately 50%) and would recommend it to other colleagues (approximately 53%). These findings, while underlining the successful implementation of The Daily Mile in the Italian school context, also point out that not all teachers would like to repeat the activity. Thus, further investigations are needed to better understand the teachers’ concerns regarding the adoption of school-based interventions such as The Daily Mile. Despite this, it is necessary to underline that our intervention included teachers who voluntary introduced The Daily Mile in their classroom, and this may have affected the results [21].

Some limitations should be underlined. A convenience sample of schools and classes was recruited for the study, the teachers voluntarily decided to introduce or not introduce The Daily Mile in their class, and therefore, no randomization was performed. Moreover, there might be some contaminations between the experimental and control groups, since, within the same school, there were classrooms that belonged to either experimental or control groups. Despite the large sample involved, future research is needed to generalise our results in Italian educational contexts, as we only recruited schools in the neighbourhood of Turin. The experimental and control groups differed in some variables at baseline; this baseline difference could make it more difficult to find any effect [15]. Additionally, the high dropout considering baseline and post-test assessment in both groups for the 6-min run test may have affected the results. The intensity during the activity was not verified (e.g., pace or distance). Thus, we were not able to quantify the effect of intensity on outcome variables. Despite this, The Daily Mile is so-called because in 15 min, 75% of the children aged 3 to 7 years and 90% of the children aged 8 to 12 years perform on the average a mile or more [22]. Furthermore, the overall physical activity of the participants was not measured, and only a few fitness tests were used. Again, the quantification of body composition was characterised by BMI and waist-to-height ratio. Other parameters on body composition should be included in future studies to better investigate this issue. However, these choices allowed us to enrol and assess a large number of students. The duration of our study was only 3 months, and the study did not include a follow-up. Future studies are needed to determine the long-term effects of this intervention better, as well as the effect of the number of sessions on physical function. Moreover, it is necessary to underline that even if the self-report survey followed the guidelines for study feasibility [25], the survey was new and original. Even if this study contributes to assessing the feasibility, safety, acceptability, and effectiveness of The Daily Mile using this program in an unexplored population, our results should be interpreted with caution.

## 5. Conclusions

In conclusion, our results showed that The Daily Mile was effective at increasing physical fitness in the Italian school contexts. Moreover, the intervention was easily and safely implemented, and it was considered by the teachers as suitable for the daily routine of Italian primary schools (children aged 6–9 years). Children spend a long time of their day at schools, and The Daily Mile might be a simple and promising program to promote a healthy lifestyle in many children.

## Figures and Tables

**Table 1:** Teachers’ survey on the feasibility of the study in terms of acceptability, perceived appropriateness, and satisfaction.

#	Question	Answers					Not Favourable	Neutral	Favourable
		1	2	3	4	5			
Section 1—Perceived positive or negative effects on participants or organisation									
1	Generally, did The Daily Mile have any positive effects inside the school environment?	0%	0%	18.8%	59.4%	21.8%	0%	18.8%	81.2%
2	Generally, did The Daily Mile have any negative effects inside the school environment?	78.1%	18.8%	0%	0%	3.1%	3.1%	0%	96.9%
3	Has The Daily Mile negatively influenced your teaching activity?	89.3%	7.1%	3.6%	0%	0%	0%	3.6%	96.4%
Section 2—Perceived appropriateness									
4	Is the activity dangerous for students’ safety?	25.0%	31.3%	34.4%	6.3%	3%	9.3%	34.4%	56.3%
5	Can The Daily Mile be easily included in the school day routine?	0%	3.1%	53.1%	21.9%	21.9%	3.1%	53.1%	43.8%
6	Was The Daily Mile appropriate for the school context?	0%	6.2%	46.9%	25.0%	21.9%	6.2%	46.9%	46.9%
7	Was The Daily Mile in accordance with the national recommendations of the curriculum?	0%	6.2%	34.4%	40.6%	18.8%	6.2%	34.4%	59.4%
8	Were there any resistances from the context (considering even parents, colleagues, executives, school staff, etc.)?	50.0%	28.1%	18.8%	3.1%	0%	3.1%	18.8%	78.1%
9	Were the students’ parents satisfied with the activity?	0%	3.1%	53.1%	40.7%	3.1%	3.1%	53.1%	43.8%
10	Did the students’ parents complain about the activity?	59.4%	28.1%	9.4%	3.1%	0%	3.1%	9.4%	87.5%
Section 3—Factors affecting easy or difficult implementation									
11	Was it stressful to perform The Daily Mile?	37.5%	53.1%	6.3%	3.1%	0%	3.1%	6.3%	90.6%
12	Was it difficult to perform The Daily Mile?	28.1%	34.4%	37.5%	0%	0%	0%	37.5%	62.5%
13	Was it easy to move the children outside the school building?	0%	0%	6.5%	54.8%	38.7%	0%	6.5%	93.5%
14	Was it easy to control the class group during the activity?	3.0%	6.3%	43.8%	37.5%	9.4%	7.3%	43.8%	46.9%
15	Was it uncomfortable to interrupt the lesson to participate in the activity?	50.0%	37.4%	6.3%	6.3%	0%	6.3%	6.3%	87.4%
16	Was it easy to restart teaching after the break?	0%	6.5%	19.4%	32.3%	41.8%	6.5%	19.4%	74.1%
Section 4—Resources needed for implementation									
17	Were the organisational aspects of the activity easy to manage?	3.1%	0%	18.8%	46.9%	31.2%	3.1%	18.8%	78.1%
18	Were many resources needed to implement The Daily Mile?	62.5%	28.1%	6.3%	3.1%	0%	3.1%	6.3%	90.6%
19	Was it easy to perform the activity independently (without external support)?	0%	18.8%	25.0%	28.1%	28.1%	18.8%	25.0%	56.2%
20	Would an external intervention be necessary for performing the activity?	34.4%	21.9%	9.4%	0%	33.3%	33.3%	9.4%	56.3%
Section 5—Satisfaction									
21	In conclusion, were you satisfied with participating in The Daily Mile?	3.1%	0%	25.0%	40.6%	31.3%	3.1%	25%	71.9%
Section 6—Intent to continue use									
22	Would you repeat The Daily Mile in the next school year?	6.3%	15.6%	28.1%	25%	25%	21.9%	28.1%	50%
23	Would you recommend The Daily Mile to colleagues of other schools?	3.1%	15.6%	28.2%	25.0%	28.1%	18.7%	28.2%	53.1%

Notes: For items 2, 3, 4, 8, 10, 11, 12, 15, 18, and 20, lower scores indicate ‘strongly agree’.

**Table 2:** Linear mixed model results of each dependent variable.

Variables	Experimental Group			Control Group			Time × Group
	Baseline	Post-Test	Change	Baseline	Post-Test	Change	
BMI (kg·m^−2^)	17.5 (17.3, 17.7)	17.4 (17.2, 176)	−0.6	17.4 (17.2, 17.7)	17.3 (17.0, 17.6)	−0.6	F = 0.792 *p* = 0.374
Waist-to-height ratio (cm)	0.46 (0.47, 0.48)	0.47 (0.46, 0.48)	2.2	0.47 (0.46, 0.47)	0.47 (0.46, 0.47)	0	F = 0.395 *p* = 0.529
6-min run test result (m)	799 (782, 816)	824 (807, 842)	3.1	848 (830, 866)	852 (834, 871)	0.5	F = 5.011 *p* = 0.026
Standing longjump result (cm)	106 (103, 109)	111 (108,114)	4.7	105 (102, 109)	109 (105, 112)	3.8	F = 3.877 *p* = 0.049

Notes: The relative percent change is the difference before and after intervention calculated on estimated mean (%). Data are presented as estimate means (95% CI). BMI, body mass index.
